# Supplementary material for: Indisulam targets RNA splicing and metabolism to serve as a therapeutic strategy for high-risk neuroblastoma
Source: Nat Commun. 2022 Mar 16;13:1380. doi: 10.1038/s41467-022-28907-3 (PMC8927615; doi:10.1038/s41467-022-28907-3)
Supplement: Supplementary file 1 — Supplementary Information [file 41467_2022_28907_MOESM1_ESM.pdf]

Supplementary figures

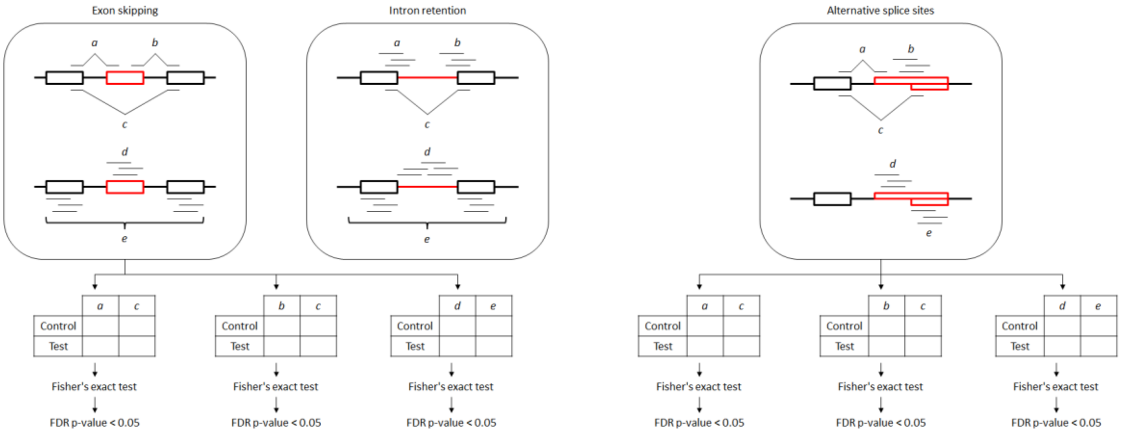

Supplementary Figure 1. A schematic overview of how Fisher's exact test was used to identify exon skipping, intron retention, and alternative splice sites. (Source https://github.com/jiwoongbio/SpliceFisher) (Han et al., 2017)

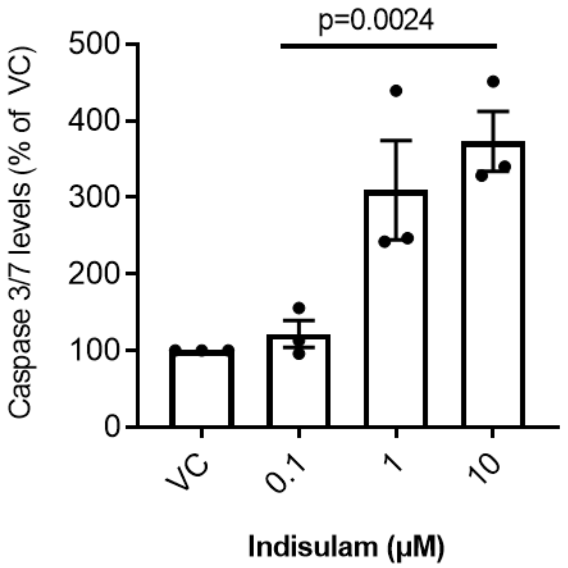

Supplementary Figure 2. Indisulam induces apoptosis in IMR-32 cells. Caspase 3/7 activity after 48 h of VC (Vehicle Control, 0.1% DMSO) or indisulam treatment at indicated doses in IMR-32 cells. Luminescence measurements were normalised to VC. Data are presented as mean values +/- SEM (*n*=3 independent experiments). Statistical significance was determined by a one-way ANOVA.

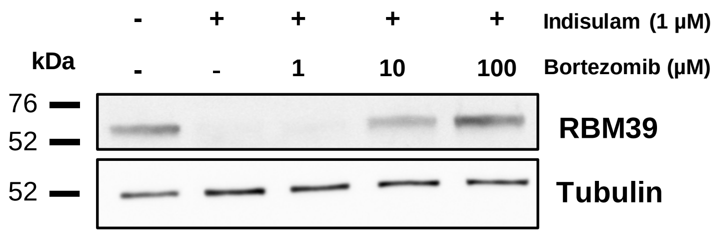

Supplementary Figure 3. RBM39 degradation was rescued with proteosome inhibitor Bortezomib. IMR-32 cells were pre-treated with indicated doses of bortezomib (proteasome inhibitor) for 2 h, followed by a 6 h treatment with indisulam (1 μM). Western blots are representative of *n*=2 independent experiments.

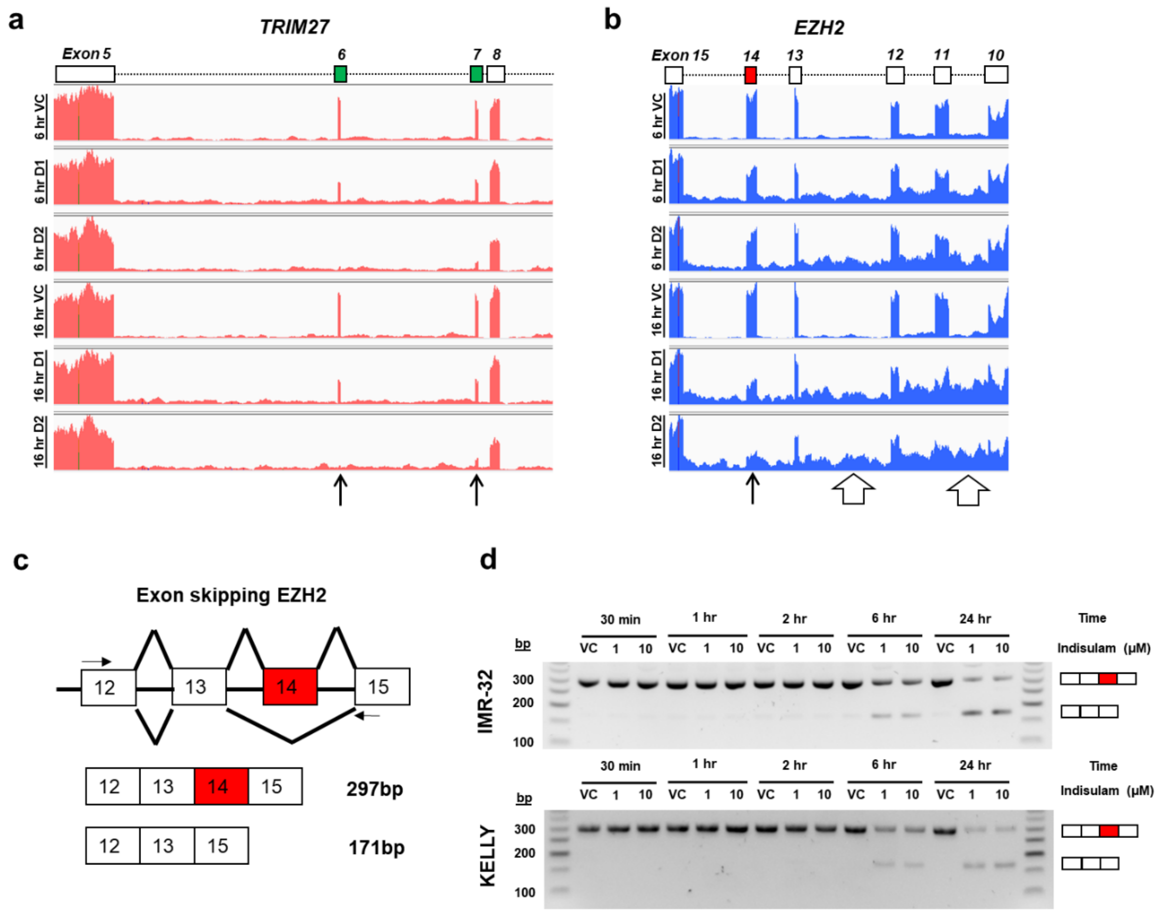

Supplementary Figure 4. Exon skipping of *TRIM27* and *EZH2*. a. RNA counts of *TRIM27* depicts loss of exon 6 and 7 post indisulam treatment, black arrows (generated with Integrative Genomes Viewer). **b.** Indisulam causes loss of exon 14, black arrow, and intron retention, white arrows in *EZH2*. **c.** Custom made primers to detect loss of exon 14 by PCR (arrows) of *EZH2*. **d**. End-point PCR of RNA post indisulam treatments for indicated time shows loss of exon 14 in both IMR-32 and KELLY neuroblastoma cell lines. VC = vehicle, D1 = 1 µM, D2 = 5 µM. RNAseq count plots generated with Integrative Genomes Viewer (Broads Institute, http://software.broadinstitute.org/software/igv/)

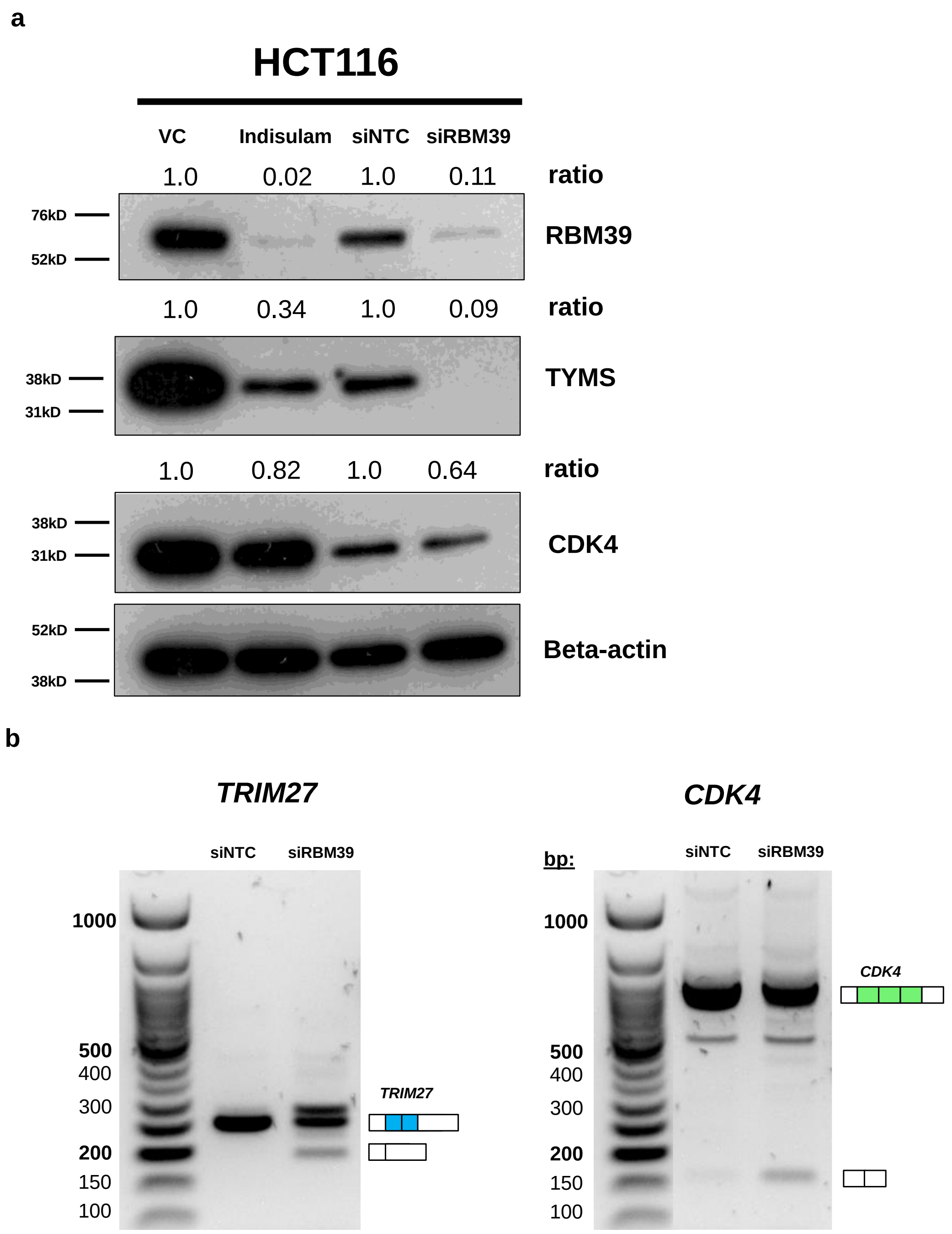

Supplementary Figure 5. Knockdown of RBM39 results in reduction of CDK4 and TYMS proteins and mis-splicing. HCT116 cells were treated with VC (Vehicle control, 0.1% DMSO) or 5 µM indisulam (16 h) or transfected with control siRNA (siNTC) or siRNA against RBM39 (siRBM39) for 72 h. Data are representative of *n*=2 independent experiments **a.** Proteins were lysed and probed for RBM39, TYMS, CDK4 and loading control (Beta-actin). Band intensities were analysed using ImageJ and represented as fold change ratios normalised to beta-actin. **b.** RNA extracted, cDNA conversion and splicing analysis of *TRIM27* and *CDK4* using PCR.

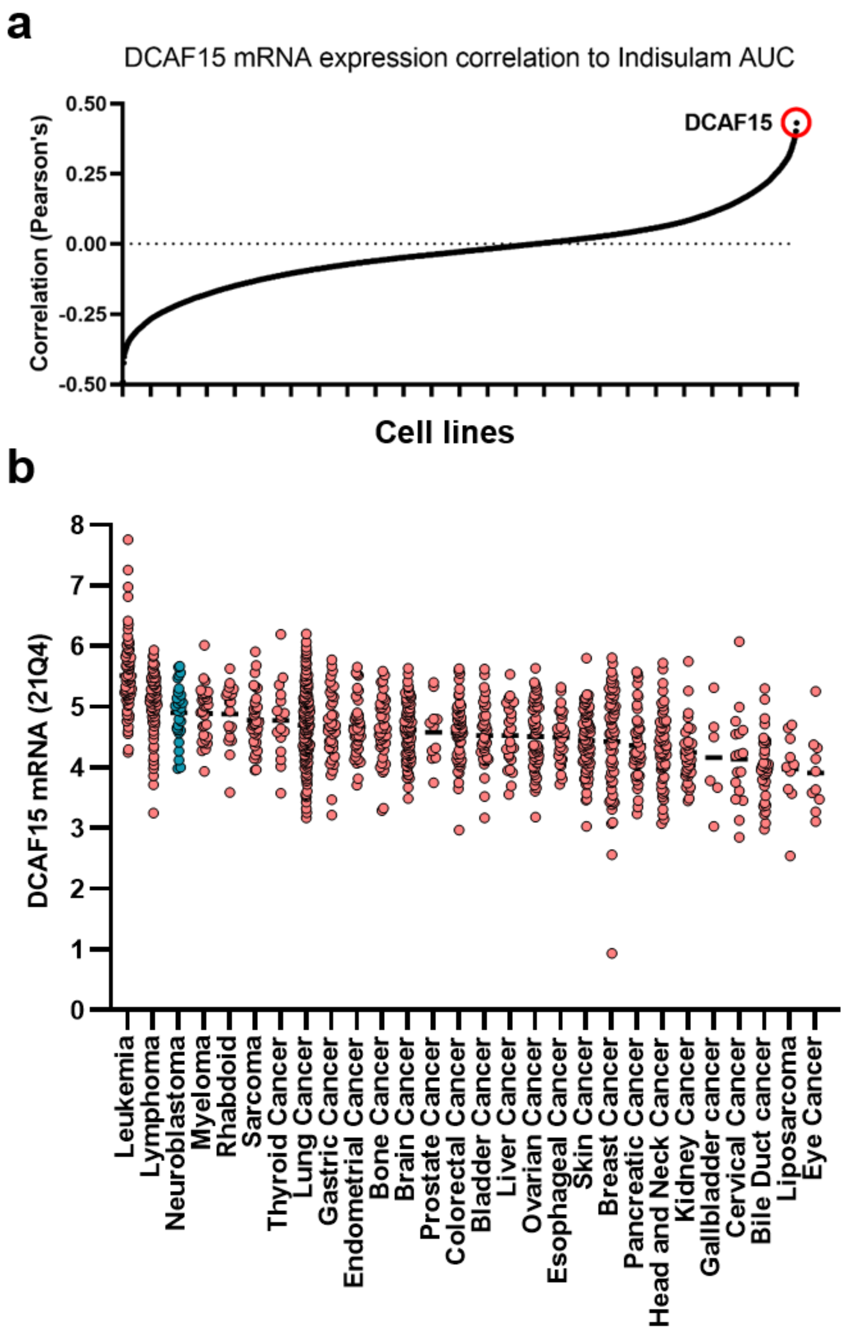

Supplementary Figure 6. a. Gene expression (Cancer Cell Line Encyclopaedia, https://portals.broadinstitute.org/ccle/home**)** plotted against sensitivity to indisulam (Area under the curve, AUC), sensitivity data was acquired from The Cancer Target Discovery and Development (CTD^2) Network (Rees et al., 2016). **b.** mRNA gene expression of *DCAF15* by RNAseq in 28 cell lineages, line indicates mean. (Expression data seq 21Q4 downloaded from https://Depmap.org/portal)

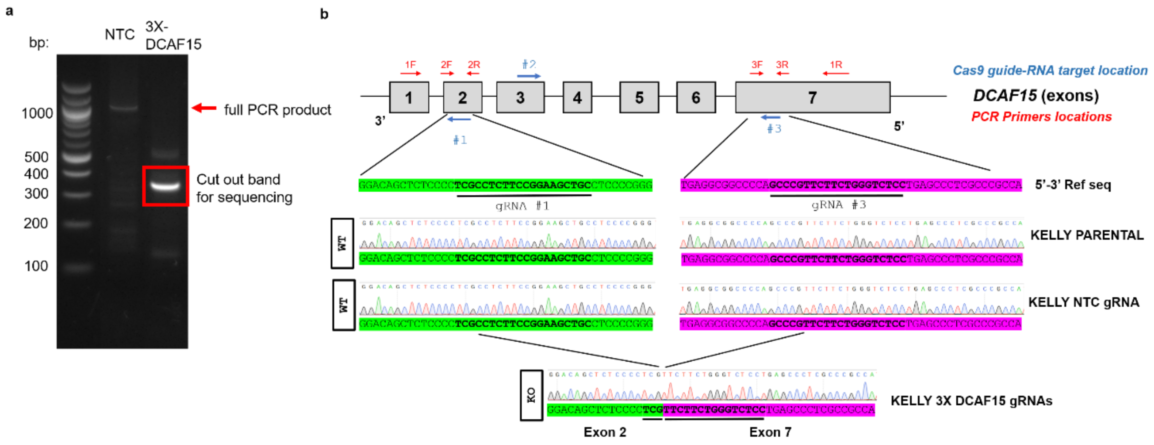

Supplementary Figure 7. CRISPR-Cas9 knockout of DCAF15 in KELLY neuroblastoma cell line. **a.** KELLY cells transfected with NTC or 3 x DCAF15 guide-RNA subjected to full-length PCR with primers 1F & 1R spanning exon 1 to 7. A significant smaller PCR product was detected for 3 x DCAF15 gRNA clone which was cut out for sequencing. **b.** DNA sequencing of DCAF15 exons 1 to 7 are shown. gRNA locations are indicated in blue and PCR primer locations in red. Sequencing showed a large deletion between Exon 2 and Exon 7 was detected, at the location of gRNA #1 and #3. Wild-type (WT) sequencing of gRNA #1 and #3 sites were confirmed with additional primer sets (gRNA #1: primers 2F & 2R, gRNA #3: primers 3F & 3R). NTC=Non-targeting control, gRNA = guideRNA

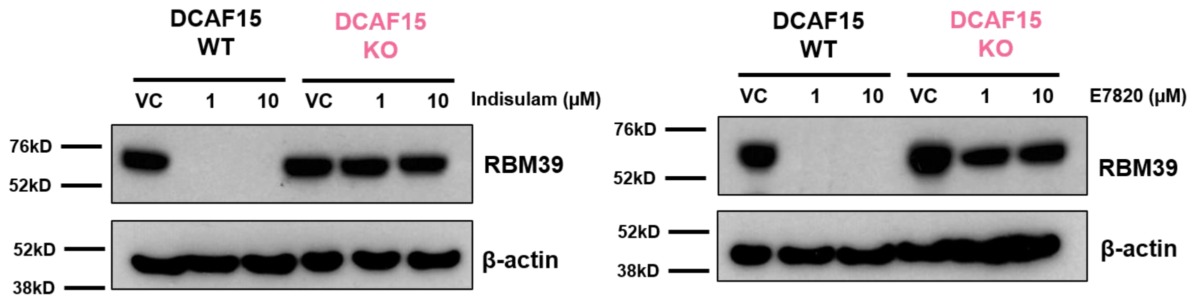

Supplementary Figure 8. DCAF15 KO rescues RBM39 degradation by indisulam or E7820. Western blot detected RBM39 and β-actin in KELLY DCAF15 WT and DCAF15 KO treated for 6 h with Indisulam or E7820 with indicated dosing. Data is a representative blot for *n*=3 independent experiments. VC = Vehicle control.

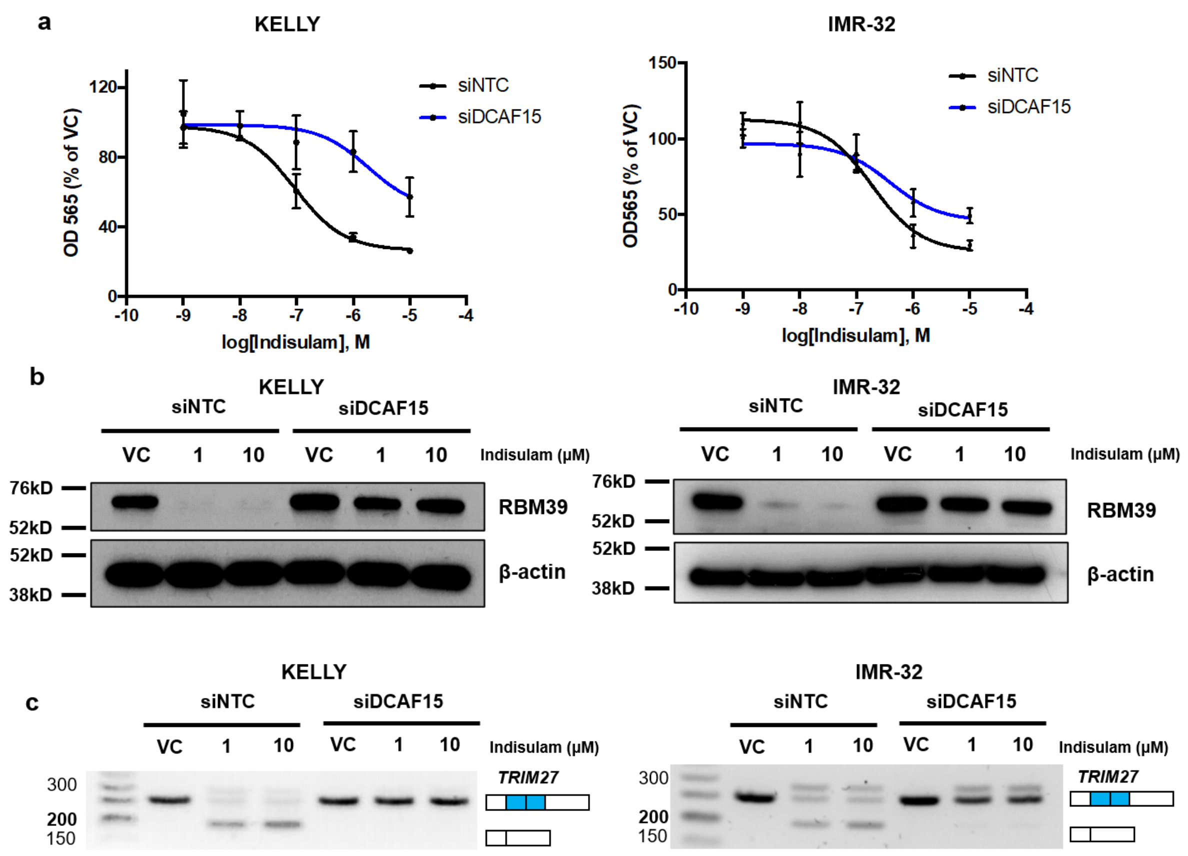

Supplementary Figure 9. Indisulam causes RBM39 degradation, cell death and mis-splicing in a DCAF15-dependent manner. a. KELLY and IMR-32 cells transfected with siNTC or siDCAF15 and treated with vehicle control (0.1% DMSO) or indisulam for 72 h and cell growth measured by SRB. Data are presented are mean +/- SD of *n*=3 independent experiments **b.** Western blot of RBM39 post indisulam treatment in KELLY and IMR-32 cells transfected with siNTC or siDCAF15. Blots are representative of *n*=2 independent experiments **c.** TRIM27 mis-splicing post indisulam treatment in KELLY and IMR-32 cells transfected with siNTC or siDCAF15. Gels are representative of *n*=2 independent experiments. siNTC = non-targeting control siRNA, VC = Vehicle control.

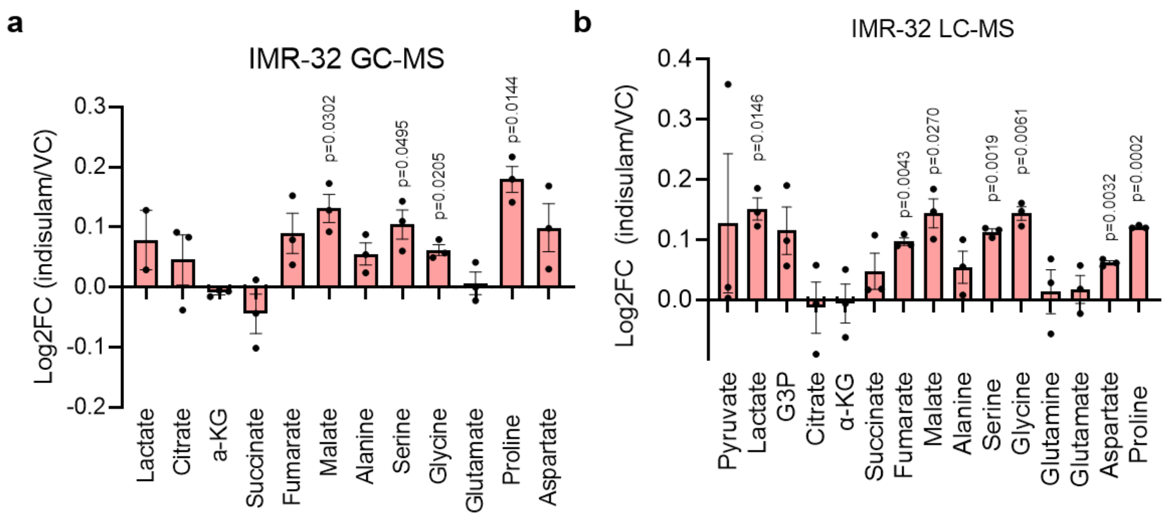

Supplementary Figure 10. Indisulam induces metabolic alterations in IMR-32 *in vitro*. **a.** GC-MS metabolic profiling in IMR-32 cells following indisulam treatment (6 h, 10µM indisulam or VC, 0.1% DMSO). Data is normalized to VC and log transformed. **b**. HILIC LC-MS/MS profiling of IMR-32 cells after treatment (6 h, 10 µM indisulam or VC, 0.1% DMSO). Data is normalised to cell number and VC and log transformed. Data are presented as mean values +/- SEM (*n*=3 independent experiments). Statistical significance was derived by a two-sided one-sample *t*-test. aKG = alpha-ketoglutarate, G3P = Glycerol-3-phosphate

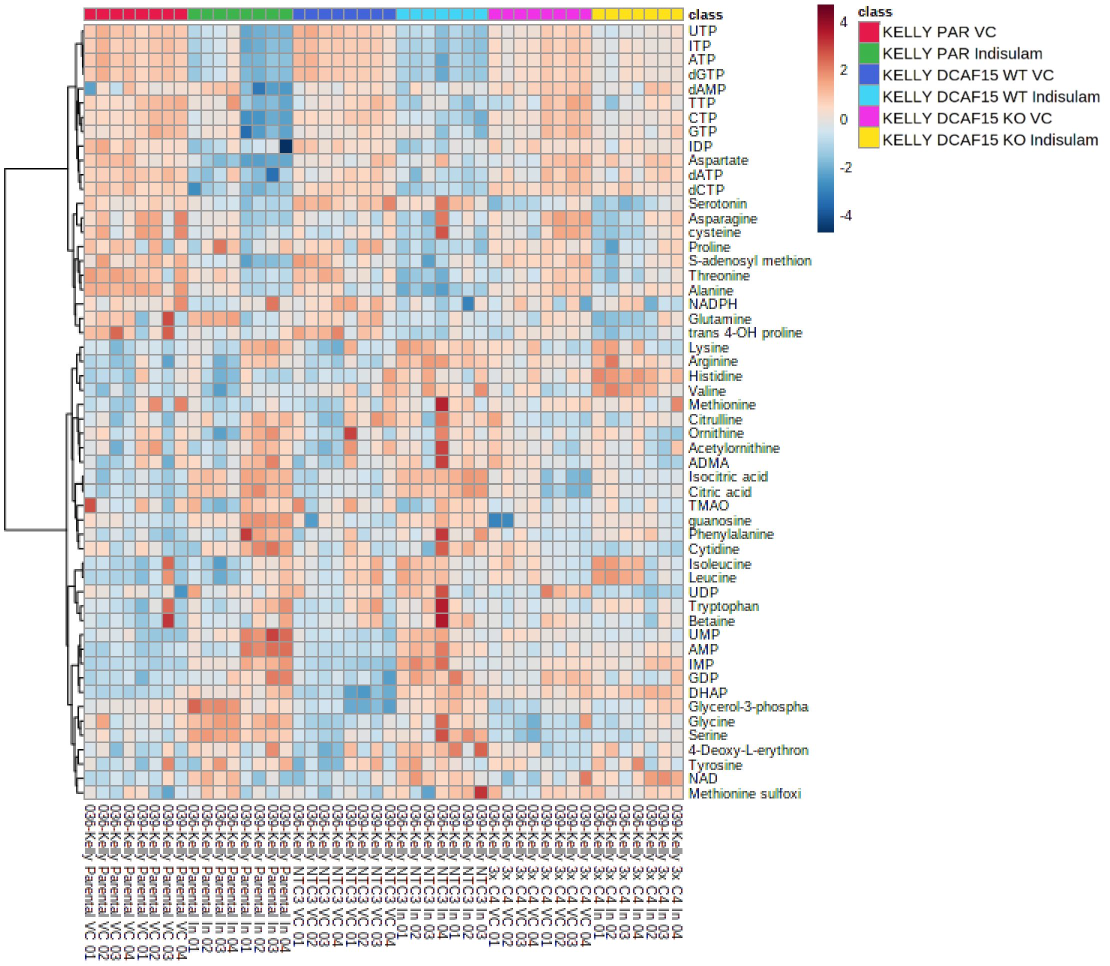

Supplementary Figure 11. Indisulam modulated global metabolism in DCAF15-dependent manner. KELLY Parental (PAR), DCAF15^{WT} or DCAF15^{KO} dosed with VC (0.1% DMSO) or 10 µM indisulam for 24 h and intracellular metabolites analysed by HILIC LC/MS. Heatmap showing hierarchal clustering of all metabolites detected in all samples (MetaboAnalyst). Data are presented in four technical replicates of *n*=2 independent experiments (Full data is in Supplementary Data File 3).

a

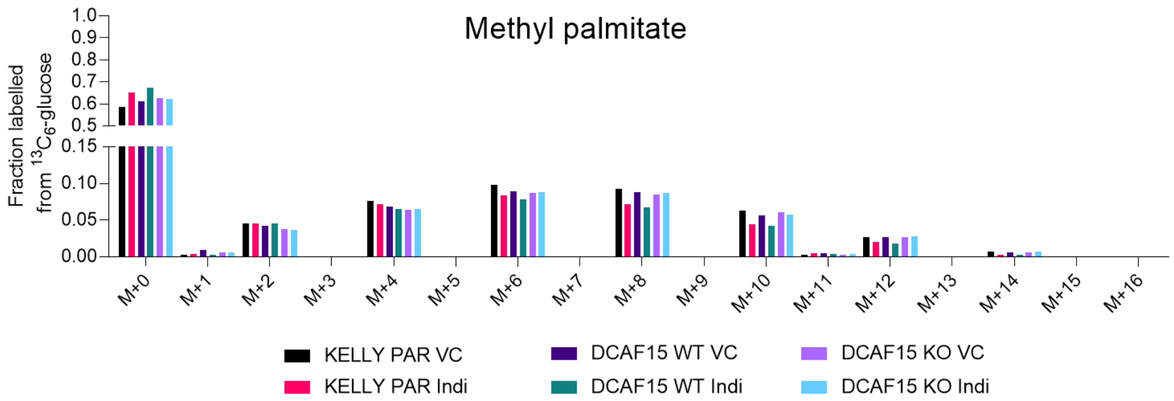

b

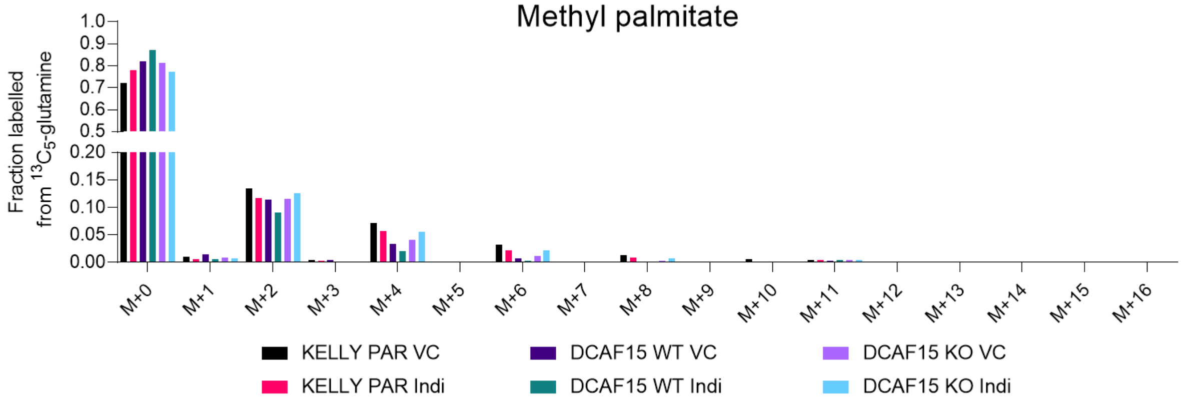

Supplementary Figure 12. Indisulam reduces carbon flux from glucose into palmitate. a-b. KELLY PARENTAL (PAR), DCAF15 WT or DCAF15 KO cells were exposed to VC (0.1% DMSO) or 10 µM indisulam in the presence of 5.6 mM $^{13}C_6$-glucose (**a**) or 2 mM $^{13}C_5$-glutamine (**b**) for 24 h. Fraction of carbons labelled from either tracer are shown. Data are presented as mean values of *n*=2 independent experiments, 3 technical replicates. VC = vehicle control, Indi = indisulam.

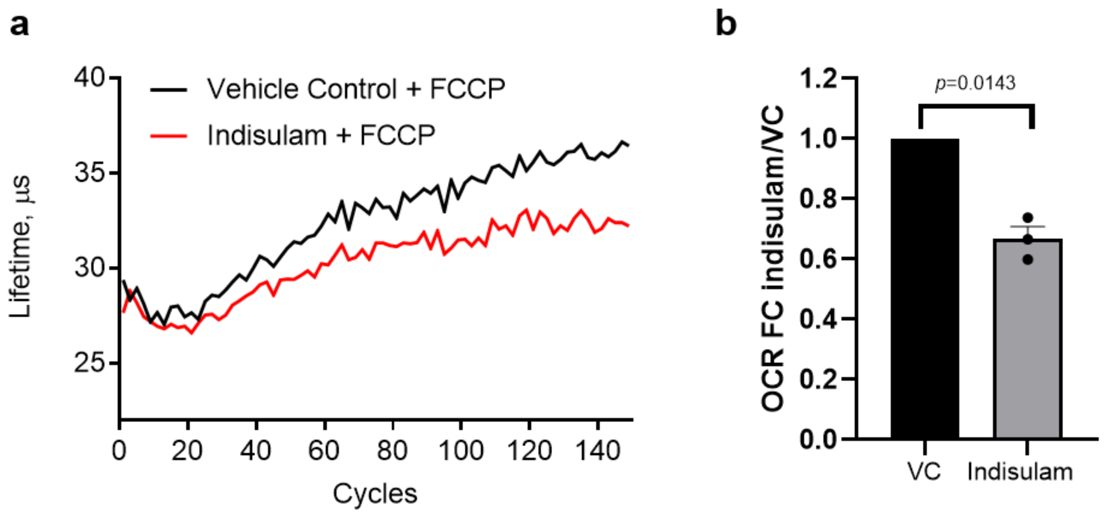

Supplementary Figure 13. Indisulam reduces cellular spare respiratory capacity. a. IMR-32 cells were treated with indisulam (10 µM) or VC (0.1% DMSO) for 2 h and then immediately treated with 2.5 µM FCCP. Fluorescence lifetime (inverse of extracellular oxygen concentration) was measured in real-time over 150 cycles (~150 min) using the MitoXpress Xtra assay (graph shown is representative of n=3 independent experiments). **b.** Fold change of oxygen consumption rate (OCR) calculated as maximum lifetime slope. Data are represented as mean values +/- SEM (n=3 independent experiments). Statistical significance was determined by a two-sided one sample t-test.

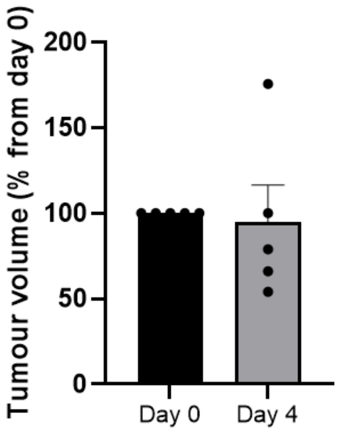

Supplementary Figure 14. Mice xenograft (IMR-32) tumour volumes on Day 4. IMR-32 xenograft tumours treated for 4 days with indisulam (25 mg kg^{-1}) were harvested for pharmacodynamic analysis. Data are presented as mean values +/- SEM.

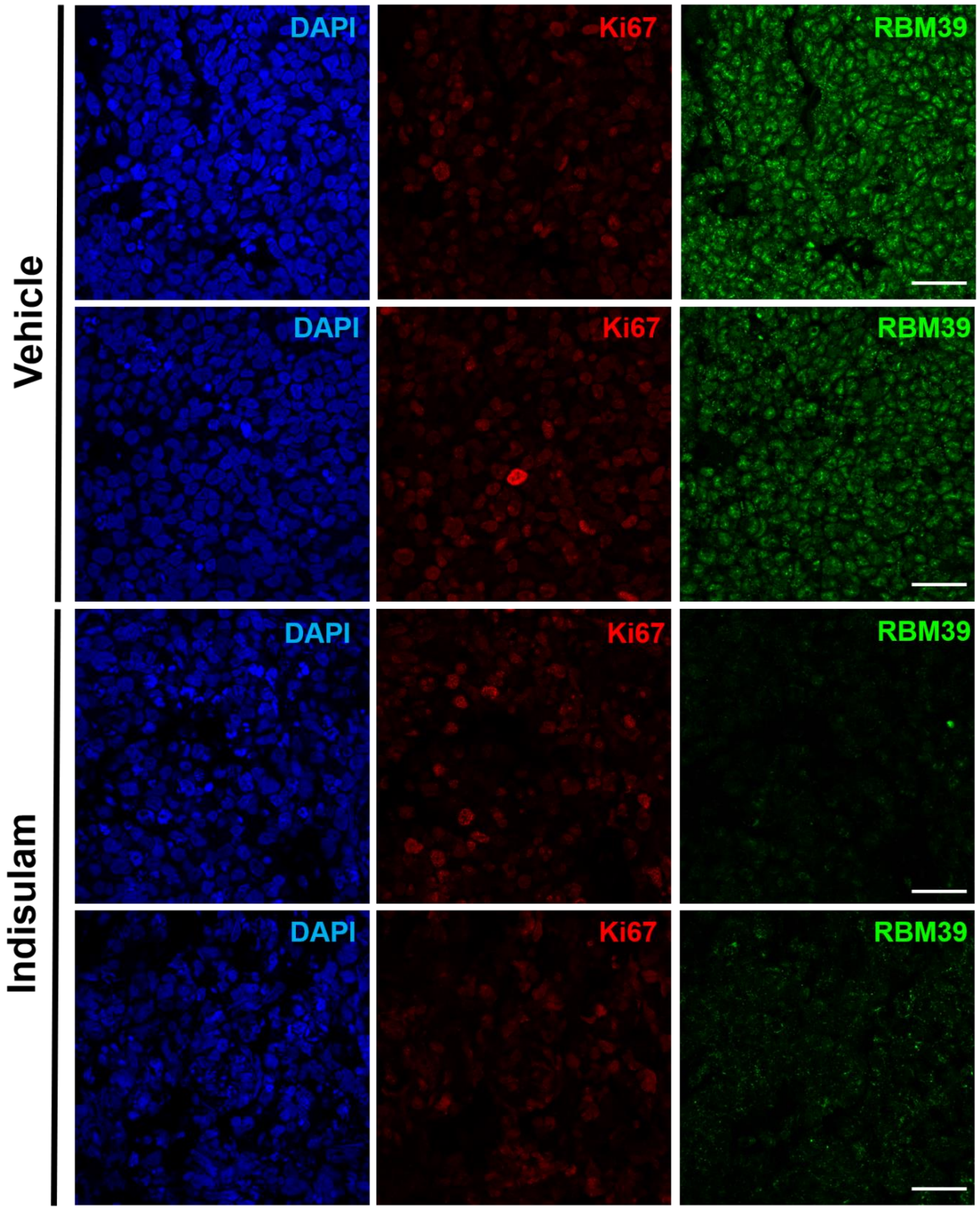

Supplementary Figure 15. Mice xenograft (IMR-32) tumours remain positive for proliferation marker Ki67 after 4 days of treatment with indisulam. IMR-32 xenografts bearing mice were treated with vehicle or Indisulam for 4 days and tissues fixed for immunofluorescence analysis. Two tumours in each arm (vehicle or indisulam 25 mg kg^{-1}) were sectioned and probed for Ki67 and RBM39. Scale bar = 20 µm.

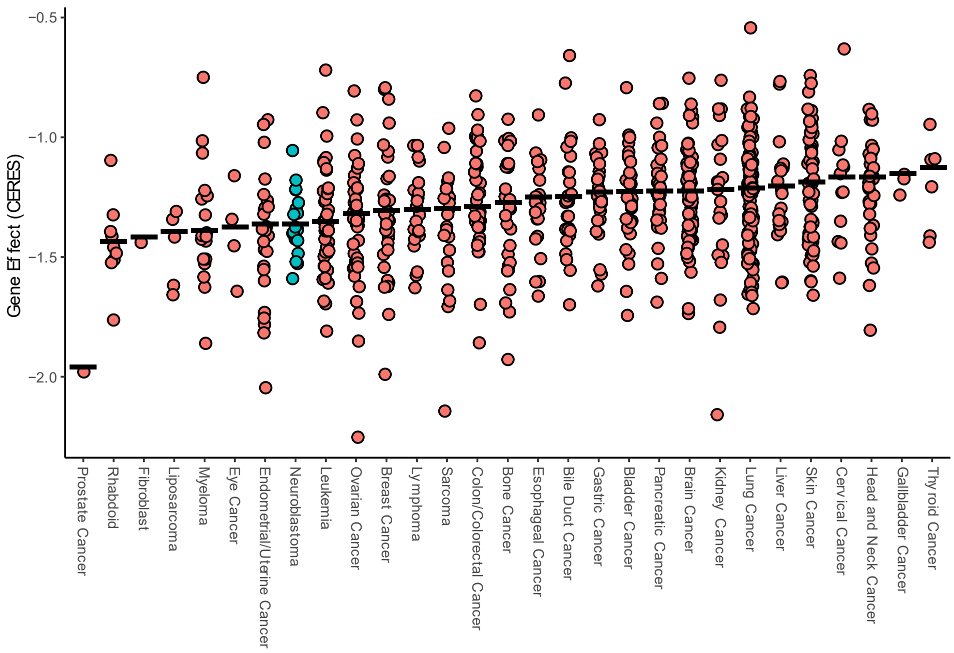

Supplementary Figure 16. RBM39 CRISPR Gene effects in cell lines from 26 tumour origins. CRISPR (Anava) Public 20Q2 data sets were downloaded from the Cancer Dependency Maps (https://depmap.org/portal/). Line indicates mean values.

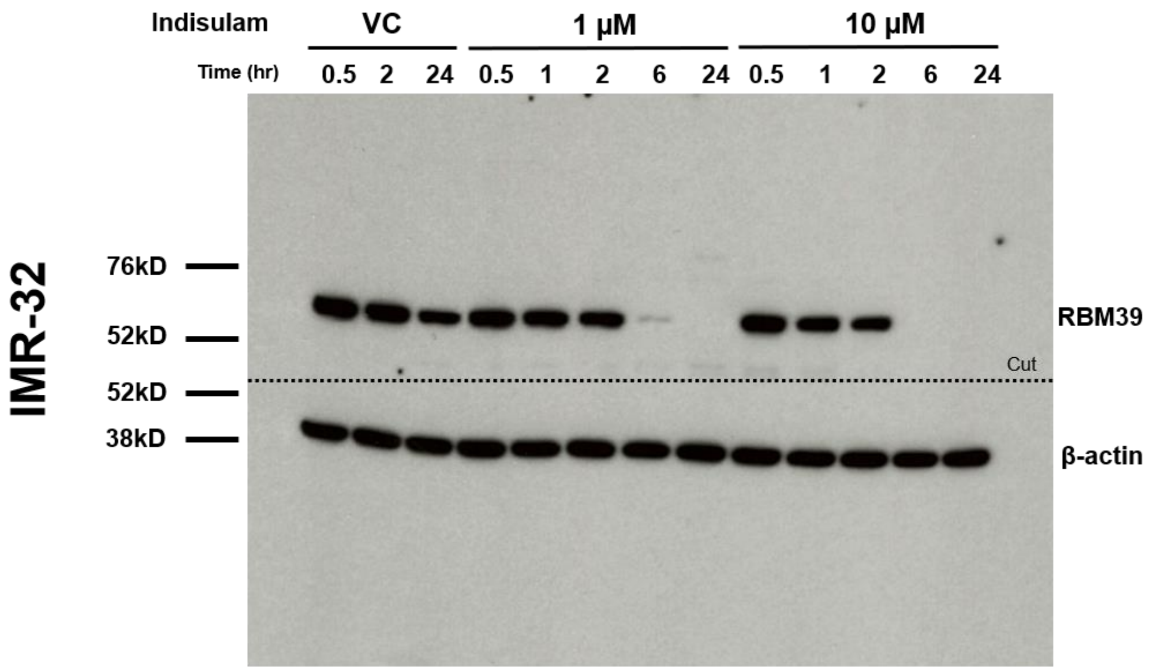

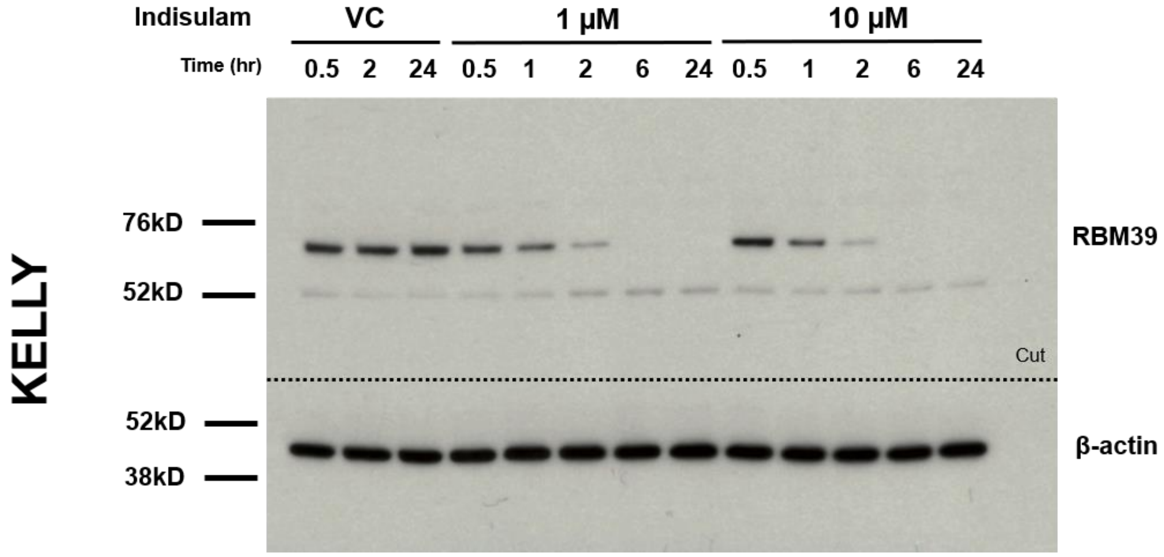

Supplementary Figure 17. Uncropped western blot images of Figure 2b.

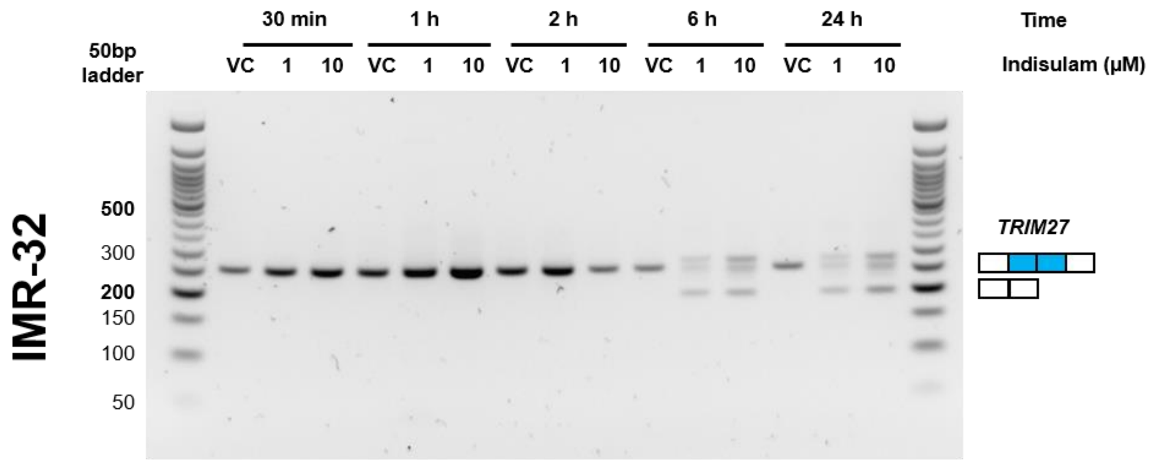

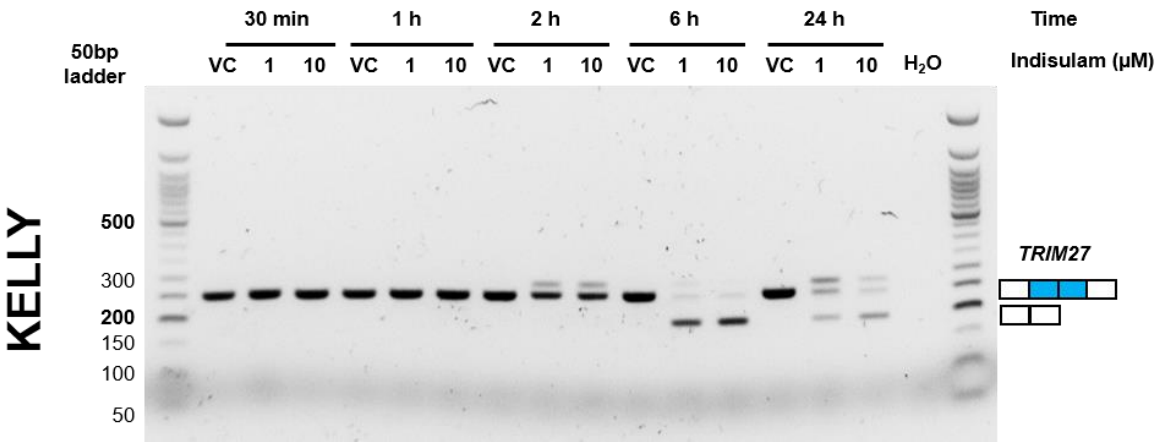

Supplementary Figure 18. Uncropped DNA gel from Figure 2f

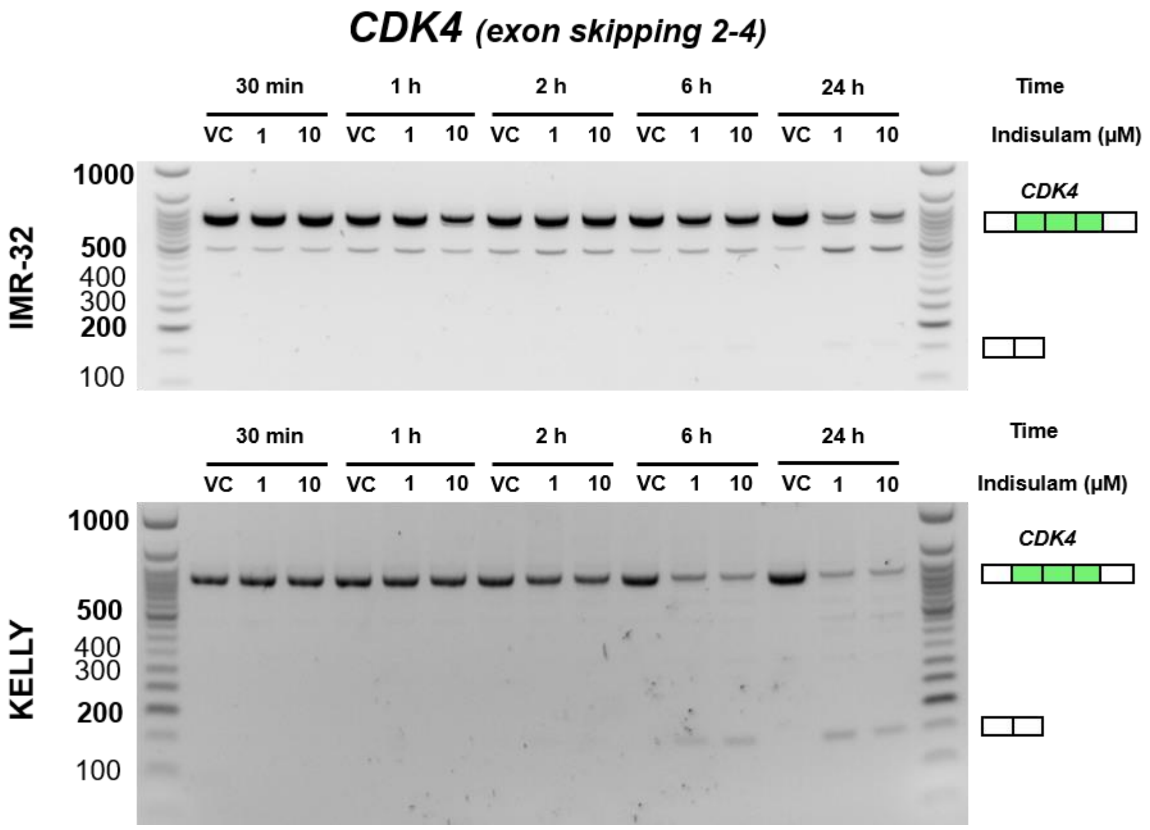

Supplementary Figure 19. Uncropped DNA gel from Figure 3e

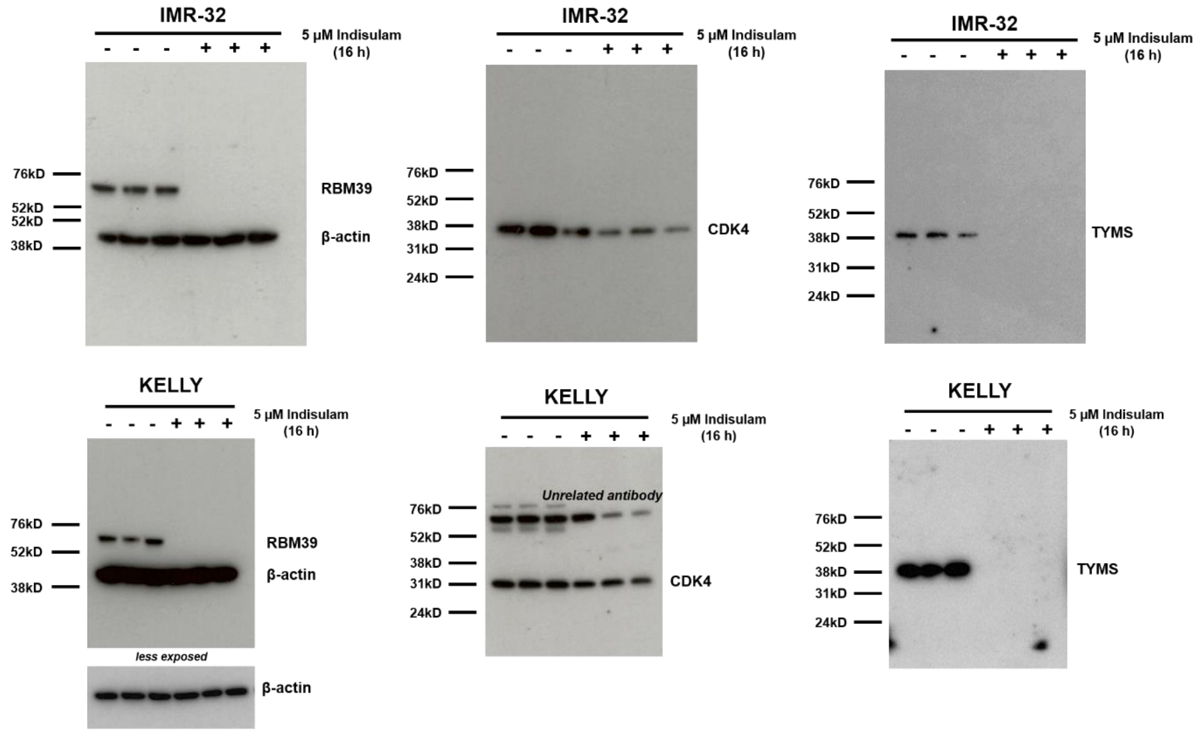

Supplementary Figure 20. Uncropped western blot for Figure 3f.

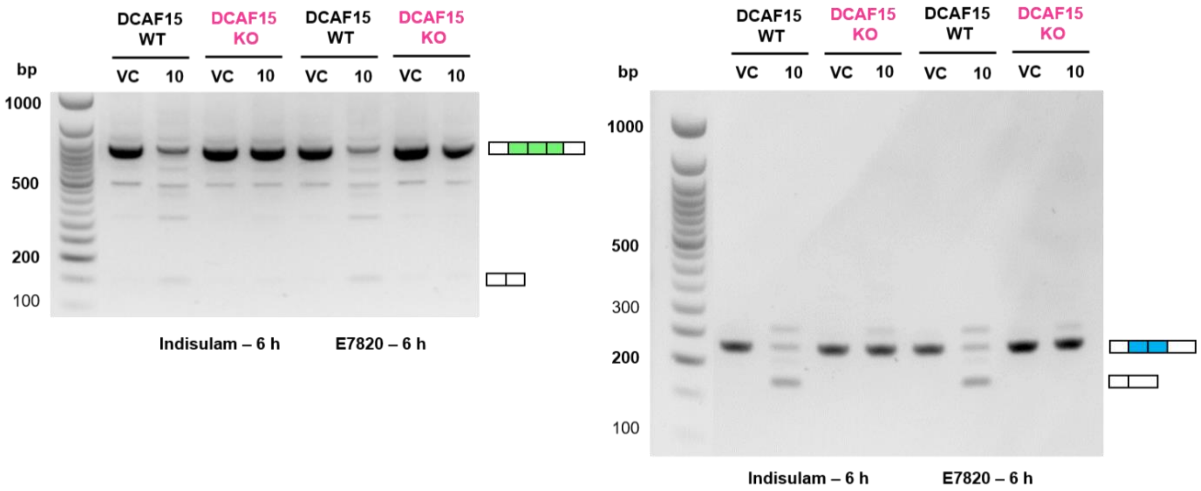

Supplementary Figure 21. Uncropped DNA gels for Figure 4f.

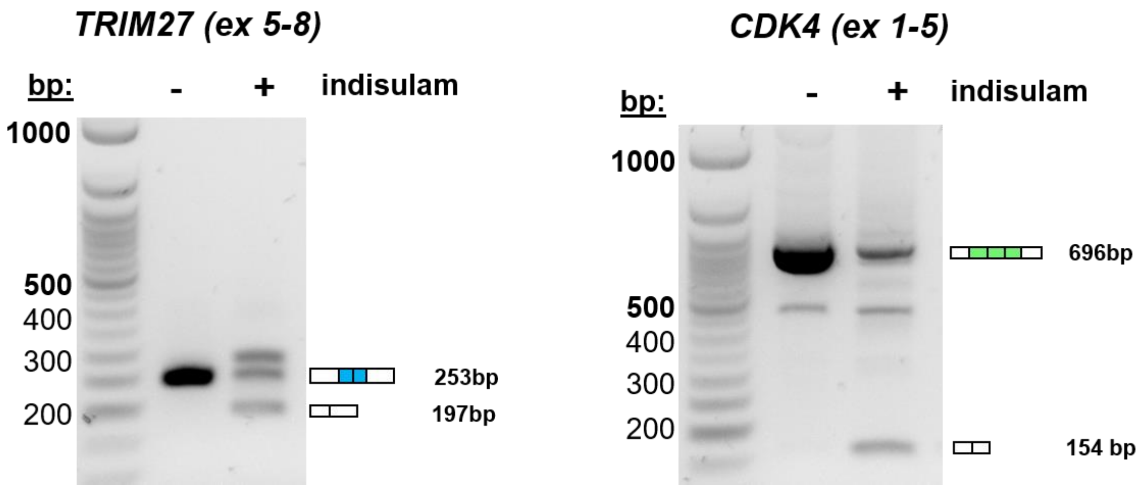

Supplementary Figure 22. Uncropped DNA gel of Figure 7f and g.

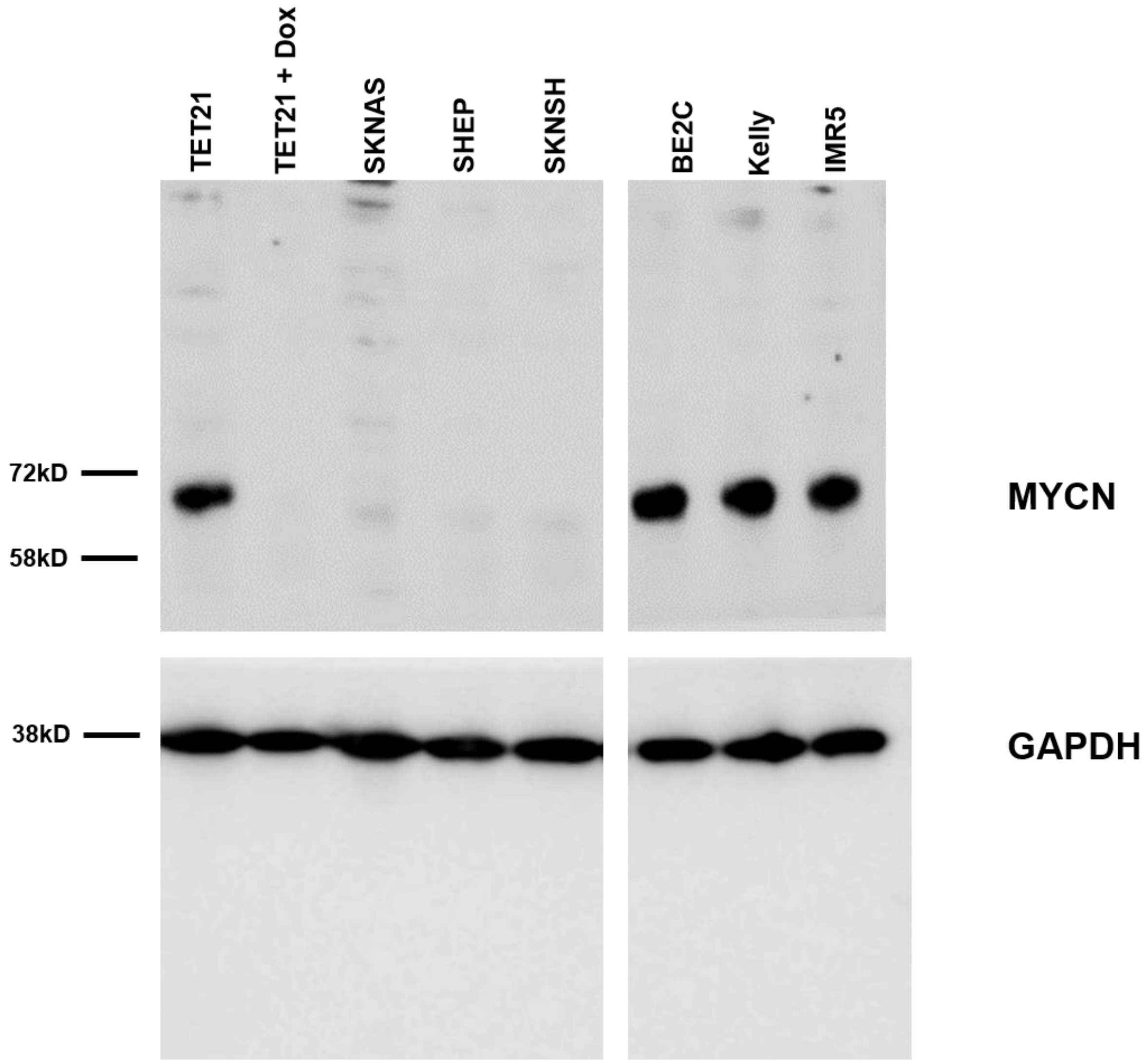

Supplementary Figure 23. Uncropped western blot for Figure 9c and d.

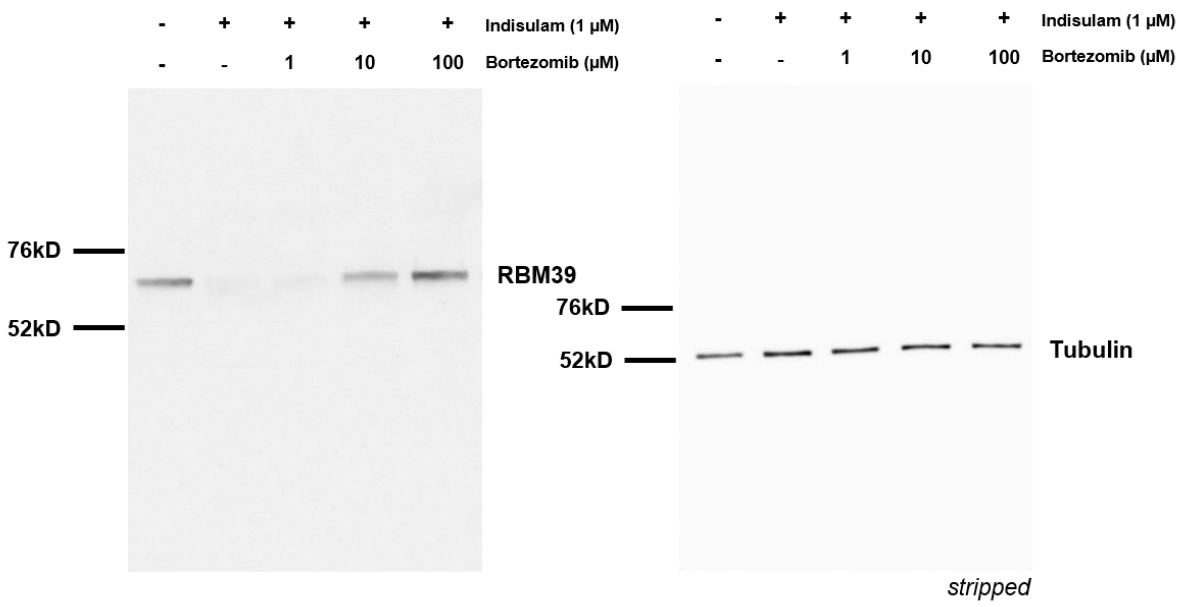

Supplementary Figure 24. Uncropped western blot for Supplementary Figure 3

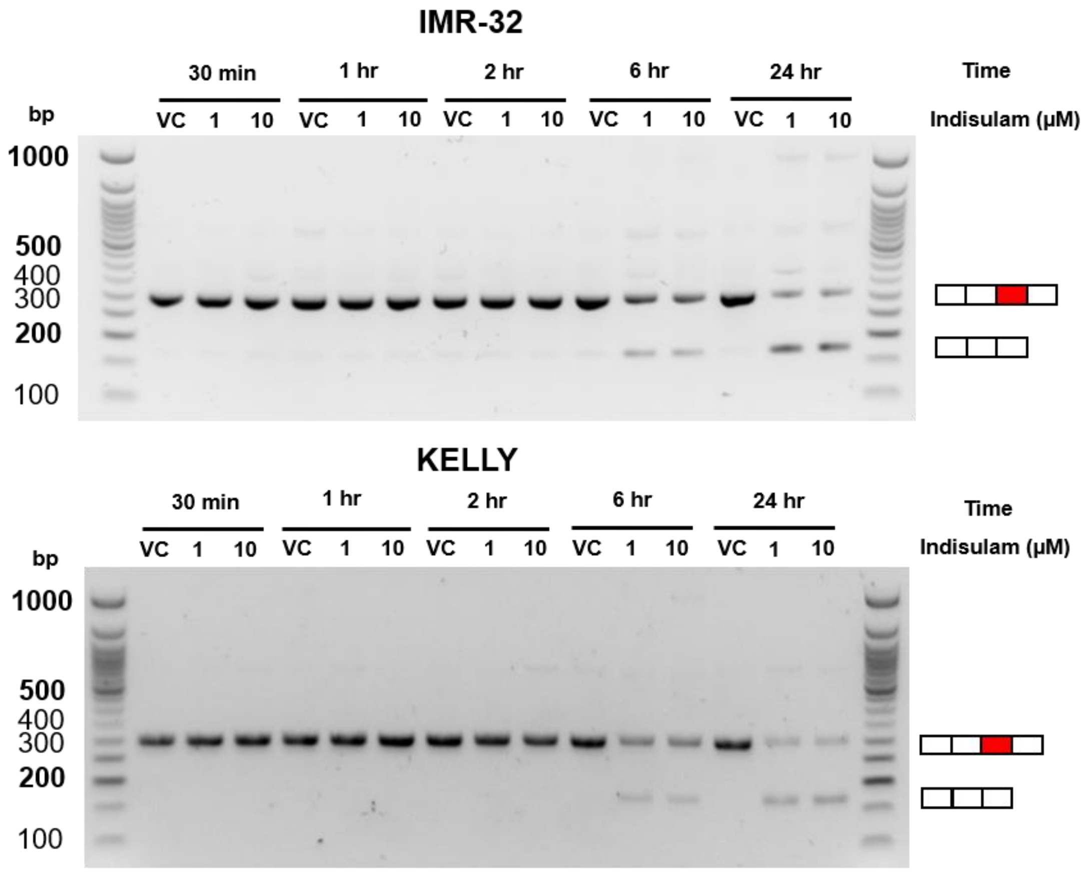

Supplementary Figure 25. Uncropped DNA gel for Supplementary Figure 4d.

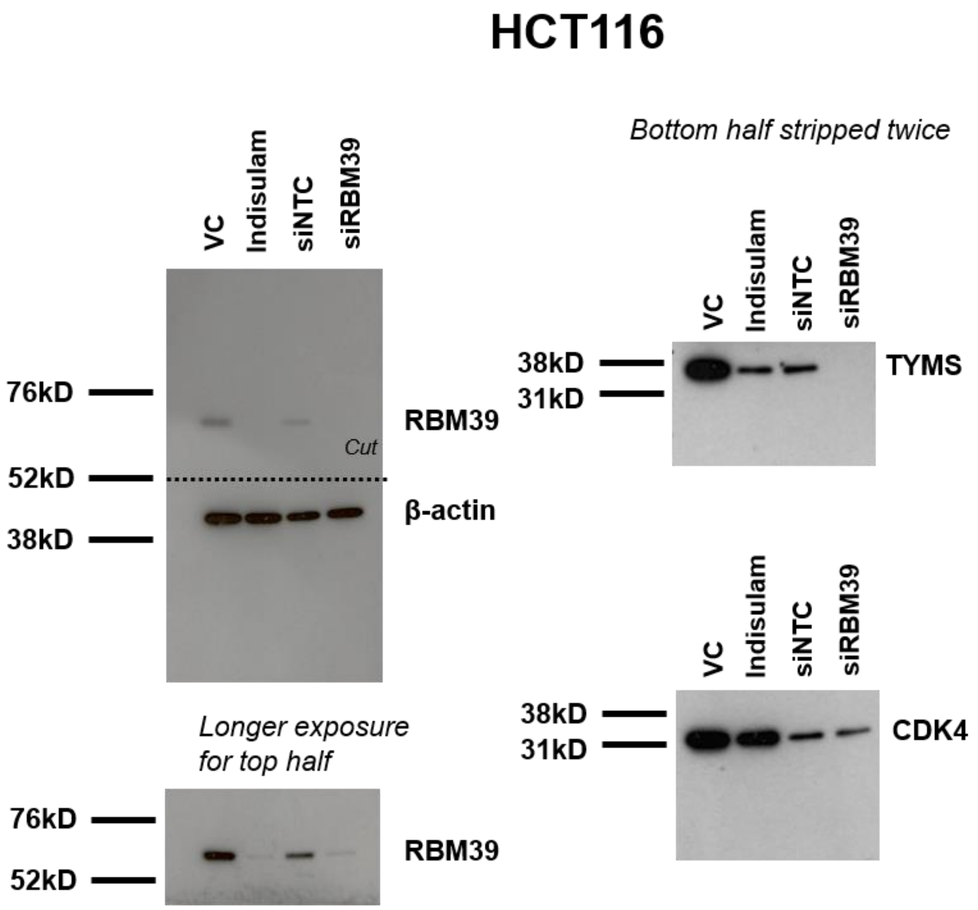

Supplementary Figure 26. Uncropped western blot for Supplementary Figure 5

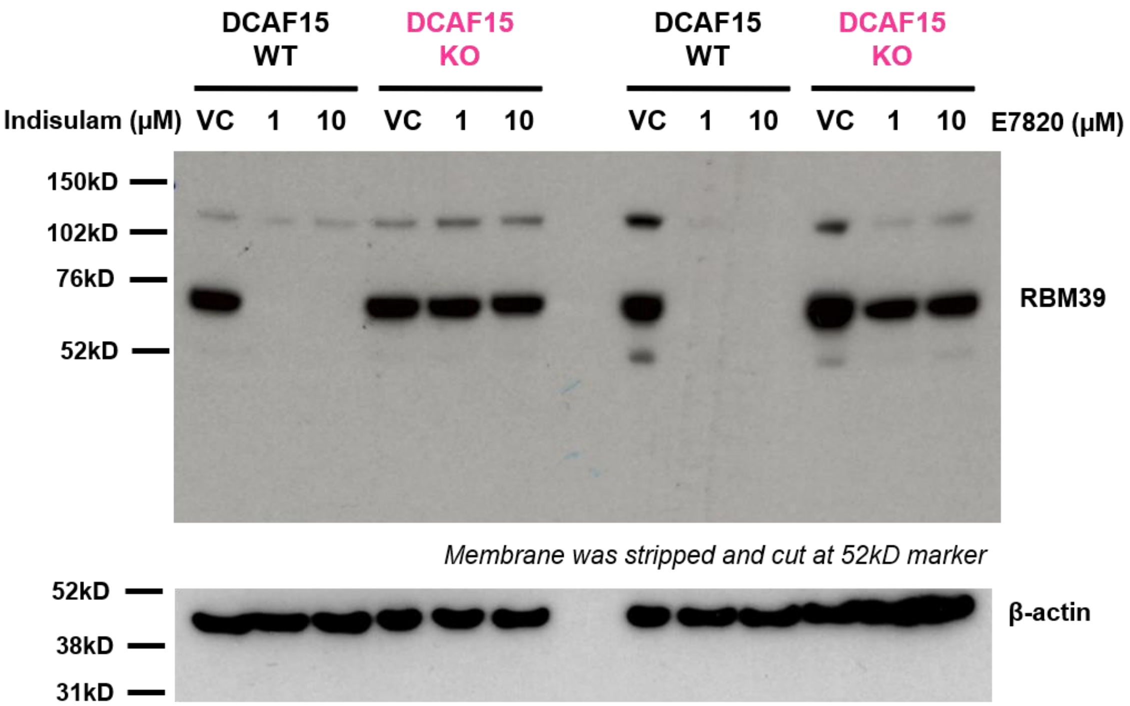

Supplementary Figure 27. Uncropped western blot for Supplementary Figure 8

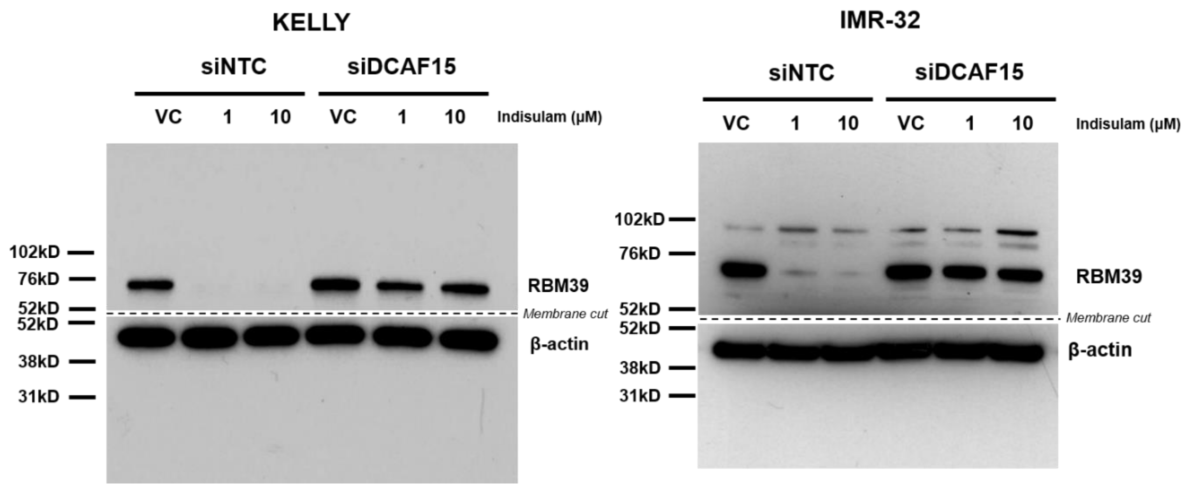

Supplementary Figure 28. Uncropped western blot for Supplementary Figure 9b.

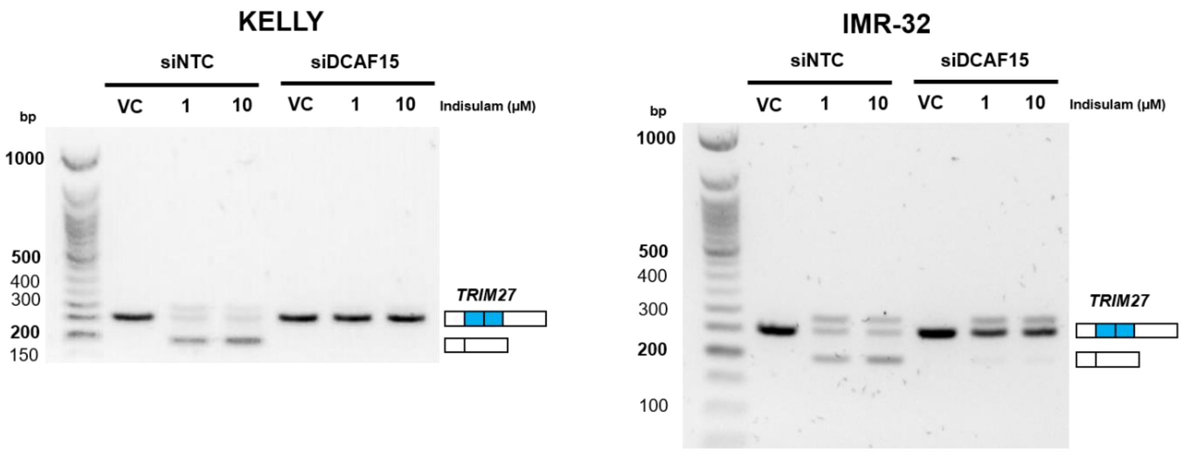

Supplementary Figure 29. Uncropped DNA gel for Supplementary Figure 9d
