# Supplementary material for: Indisulam targets RNA splicing and metabolism to serve as a therapeutic strategy for high-risk neuroblastoma
Source: Nat Commun. 2022 Mar 16;13:1380. doi: 10.1038/s41467-022-28907-3 (PMC8927615; doi:10.1038/s41467-022-28907-3)
Supplement: Supplementary file 3 — Description of Additional Supplementary Files [file 41467_2022_28907_MOESM3_ESM.pdf]

Description of Additional Supplementary Files

File Name: Supplementary Data 1
Description: Pathway enrichment analysis on RNA splicing and proteomics analysis

File Name: Supplementary Data 2
Description: 13Cisotope labelling data in DCAF15 WT and KO KELLY cells

File Name: Supplementary Data 3
Description: LC-MS metabolomic analysis in DCAF15 WT and KO KELLY cells

File Name: Supplementary Data 4
Description: LC-MS metabolomic analysis in IMR-32 xenograft tissue
