# Supplementary material for: Indisulam targets RNA splicing and metabolism to serve as a therapeutic strategy for high-risk neuroblastoma
Source: Nat Commun. 2022 Mar 16;13:1380. doi: 10.1038/s41467-022-28907-3 (PMC8927615; doi:10.1038/s41467-022-28907-3)
Supplement: Supplementary file 8 — Reporting Summary [file 41467_2022_28907_MOESM8_ESM.pdf]

nature portfolio

Corresponding author(s): Hector Keun

Last updated by author(s): 18-01-2022

Reporting Summary

Nature Portfolio wishes to improve the reproducibility of the work that we publish. This form provides structure for consistency and transparency in reporting. For further information on Nature Portfolio policies, see our Editorial Policies and the Editorial Policy Checklist.

Statistics

For all statistical analyses, confirm that the following items are present in the figure legend, table legend, main text, or Methods section.

n/a	Confirmed	
☐	☒	The exact sample size (*n*) for each experimental group/condition, given as a discrete number and unit of measurement
☐	☒	A statement on whether measurements were taken from distinct samples or whether the same sample was measured repeatedly
☐	☒	The statistical test(s) used AND whether they are one- or two-sided<br>*Only common tests should be described solely by name; describe more complex techniques in the Methods section.*
☐	☒	A description of all covariates tested
☐	☒	A description of any assumptions or corrections, such as tests of normality and adjustment for multiple comparisons
☐	☒	A full description of the statistical parameters including central tendency (e.g. means) or other basic estimates (e.g. regression coefficient) AND variation (e.g. standard deviation) or associated estimates of uncertainty (e.g. confidence intervals)
☐	☒	For null hypothesis testing, the test statistic (e.g. *F*, *t*, *r*) with confidence intervals, effect sizes, degrees of freedom and *P* value noted<br>*Give P values as exact values whenever suitable.*
☒	☐	For Bayesian analysis, information on the choice of priors and Markov chain Monte Carlo settings
☒	☐	For hierarchical and complex designs, identification of the appropriate level for tests and full reporting of outcomes
☒	☐	Estimates of effect sizes (e.g. Cohen's *d*, Pearson's *r*), indicating how they were calculated

Our web collection on statistics for biologists contains articles on many of the points above.

Software and code

Policy information about availability of computer code

Data collection	No software was used for data collection
Data analysis	The following software was used for data analysis. Data analysis was performed on Graphpad PRISM (V9.2.0) and R package Rsubread (v 1.34.7). Incucyte Zoom software (v2020B), CLARIOstar MARS Software (v3.40), Image J (v1.52), Horos medical image viewer (v3.3.6). Zen 2009 (black version), MaxQuant software platform (v1.6.1.0), MetaboAnalyst (v5.0)

For manuscripts utilizing custom algorithms or software that are central to the research but not yet described in published literature, software must be made available to editors and reviewers. We strongly encourage code deposition in a community repository (e.g. GitHub). See the Nature Portfolio guidelines for submitting code & software for further information.

Data

Policy information about availability of data

All manuscripts must include a data availability statement. This statement should provide the following information, where applicable:

- Accession codes, unique identifiers, or web links for publicly available datasets
- A description of any restrictions on data availability
- For clinical datasets or third party data, please ensure that the statement adheres to our policy

The mass spectrometry proteomics data generated in this study have been deposited to the ProteomeXchange Consortium via the PRIDE49 partner repository under the accession codePXD022164 [https://www.ebi.ac.uk/pride/archive/projects/PXD022164] . The RNAseq data generated in this study have been deposited in NCBI's Gene Expression Omnibus50 under the accession number GSE160446 [https://www.ncbi.nlm.nih.gov/geo/query/acc.cgi?acc=GSE160446]. Processed metabolome data generated in this study are available as Supplementary Data files. CERES CRISPR and CTRP data were downloaded from DepMap database (https://depmap.org/portal/). DCAF15 expression data from cancer cell lineages were downloaded from CCLE RNA-seq (https://portals.broadinstitute.org/ccle).

Field-specific reporting

Please select the one below that is the best fit for your research. If you are not sure, read the appropriate sections before making your selection.

☒ Life sciences ☐ Behavioural & social sciences ☐ Ecological, evolutionary & environmental sciences

For a reference copy of the document with all sections, see nature.com/documents/nr-reporting-summary-flat.pdf

Life sciences study design

All studies must disclose on these points even when the disclosure is negative.

Sample size	In vivo sample size (mice per group) were chosen based on previous literature, in-house in vitro experimental data and experience
Data exclusions	In the LC-MS/MS metabolomics of KELLY cells, Dixon's Q test was applied to two samples with a suspected technical error. Results indicated that 80% (039-Kelly DCAF15 WT Indisulam 04) and 98% (039-Kelly DCAF15 KO Indisulam 01) of the metabolites analyzed had outlier values with a 95% confidence interval according to Dixon's Q test (RB Dean and WJ Dixon "Simplified Statistics for Small Numbers of Observations". Anal. Chem., 1951, 23 (4), 636–638.).
Replication	Experiments were conducted 3 times for a single output, unless multiple time points and dosages were generated in one experiment, then these were conducted twice (for example, Figure 2). Information on replication of each experiment is stated in the legend.
Randomization	In both in vivo models mice were randomly enrolled in treatment or control groups
Blinding	Animals were blinded from the investigators through standard protocols at ICR

Reporting for specific materials, systems and methods

We require information from authors about some types of materials, experimental systems and methods used in many studies. Here, indicate whether each material, system or method listed is relevant to your study. If you are not sure if a list item applies to your research, read the appropriate section before selecting a response.

Materials & experimental systems

n/a	Involved in the study	
☐	☒	Antibodies
☐	☒	Eukaryotic cell lines
☒	☐	Palaeontology and archaeology
☐	☒	Animals and other organisms
☒	☐	Human research participants
☒	☐	Clinical data
☒	☐	Dual use research of concern

Methods

n/a	Involved in the study	
☒	☐	ChIP-seq
☒	☐	Flow cytometry
☒	☐	MRI-based neuroimaging

Antibodies

Antibodies used	anti-RBM39 (HPA001591, Sigma, 1:1000), anti-beta-actin (ab8226, Abcam, 1:10000), anti-CDK4 (ab108357, Abcam, 1:1000), anti-TYMS (ab108995, Abcam, 1:1000), anti-n-Myc (OP13, Merckmillipore, 1:1000) and Anti-GAPDH (2118, Cell Signaling, 1:5000), Ki67 (Cat #556003, BD Bioscience, 1:1000)
Validation	Antibodies were selected from respectable sources and highly cited in the literature. Details on validation for each antibody is below.<br>RBM39: validated by Sigma's "Antibody Enhanced Validation methodoloy" through RNAi knockdown. Antibody was also validated by siRNA knockdown in our laboratory.<br>beta-actin: 5-star reviewed and extremely highly cited (2331) validated antibody for us as loading control.<br>CDK4: 5-star reviewed and highly cited (113) antibody. Validated by Abcam through knockout<br>TYMS: 4.5/5 star reviewed and cited (12) antibody. Validated by Abam through knockout.<br>n-MYC: highly cited antibody. Validated in house on IMR5 cell line as recommended by supplier.<br>GAPDH: highly cited (5350) validated antibody for use as loading control<br>Ki67: highly cited (223) and validated by Milipore

Eukaryotic cell lines

Policy information about cell lines

Cell line source(s)	IMR-32, BE(2)C and SK-N-AS cells cell were purchased from the American Type Culture Collection (ATCC) (Manassas, VA). KELLY and SH-SY-5Y were obtained from the German Collection of Microorganisms and Cell Culture (DSMZ, Germany). SK-N-

	SH was purchased from the Human Protein Atlas project (HPA). IMR-5 and Tet21 were a kind gift from Eilers lab, University of Wurzburg and SHEP cells were a kind gift from Weiss lab, University of California at San Francisco
Authentication	All cell lines used in this study were authenticated through short tandem repeats
Mycoplasma contamination	All cell lines tested negative for mycoplasma and were regularly tested
Commonly misidentified lines (See ICLAC register)	No commonly misidentified lines were used in this study

Animals and other organisms

Policy information about studies involving animals; ARRIVE guidelines recommended for reporting animal research

Laboratory animals	Xenograft studies were performed in NCr-Foxn1nu mice. Mice were all female, aged 6 weeks and weigh edon average 24.6g. Th-MYCN transgene studies were performed with 129X1/SvJ-Tg(Th-MYCN)41Waw/Nci mice. Mice were both male and female, aged 71 days +/- 12 days and weighed on average 24.3g. Rooms were maintained between 20 and 24 degrees Celsius. Humidity in the rooms were kept between 45-50% and a 12:12 day/night cycle was applied.
Wild animals	No wild animals were used in this study
Field-collected samples	No field samples were collected in this study
Ethics oversight	All experimental protocols were approved and monitored by The Institute of Cancer Research Animal Welfare and Ethical Review Body (PPL no. 70/7945, later PPL P91E52C32), in compliance with the UK Home Office Animals (Scientific Procedures) Act 1986, the United Kingdom National Cancer Research Institute guidelines for the welfare of animals in cancer research and the ARRIVE guidelines.

Note that full information on the approval of the study protocol must also be provided in the manuscript.
